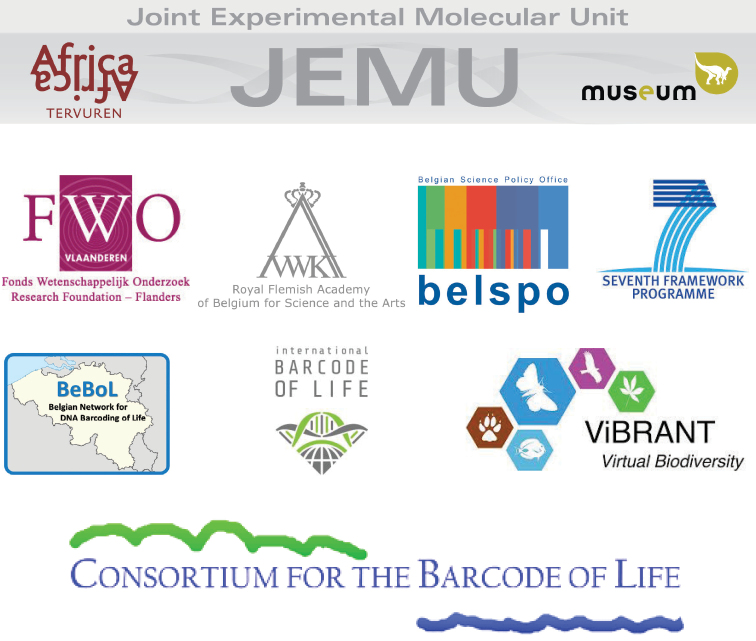# Editorial

**DOI:** 10.3897/zookeys.365.6681

**Published:** 2013-12-30

**Authors:** Zoltán T Nagy, Thierry Backeljau, Marc De Meyer, Kurt Jordaens

**Affiliations:** 1 Royal Belgian Institute of Natural Sciences, OD Taxonomy and Phylogeny (JEMU), Vautierstraat 29 - B-1000 Brussels, Belgium; 2 Evolutionary Ecology Group, University of Antwerp, Groenenborgerlaan 171 - B-2020 Antwerp, Belgium; 3 Royal Museum for Central Africa (JEMU), Leuvensesteenweg 13 - B-3080 Tervuren, Belgium

Since its formal introduction in 2003, DNA barcoding has become a well-accepted and popular tool for the identification of species and the detection of cryptic taxonomic diversity. Hence, it is not surprising that the past decade has witnessed a boom of DNA barcoding studies, up to the point that currently the method is becoming an integral part of taxonomic practice. This does not mean that DNA barcoding is some sort of magic technology, capable of solving all taxonomic problems. Such a view would indeed be simplistic and, in fact, was never claimed by the DNA barcoding community. Instead, DNA barcoding is a practical tool that facilitates species (or more generally, taxon) identification, without solving or considering the central taxonomic question as to what a species really is. Yet, being primarily an identification tool, DNA barcoding has a tremendous potential for a wide variety of possible applications. This point is globally well-recognized and hence, after the foundation of the overarching, worldwide International Barcode of Life Project (iBOL) and the Consortium for the Barcode of Life (CBOL), which initiated several taxon, regional or problem-oriented DNA barcoding initiatives, several countries, institutions and organizations have joined these international bodies and launched their own national or regional projects.

Also Belgium created its DNA barcoding consortium, the Belgian Network for DNA Barcoding, which embodies the Belgian Barcoding of Life (BeBoL) initiative. This network was established in January 2011 with the financial support of the Fund for Scientific Research – Flanders (FWO). It is financially administrated by the University of Antwerp, but its activities are coordinated by the Joint Experimental Molecular Unit (JEMU), a molecular systematics research facility shared by the Royal Museum for Central Africa (RMCA) and the Royal Belgian Institute of Natural Sciences (RBINS), and financed by the Belgian Science Policy Office (BELSPO). It currently involves 23 Belgian members, including not only federal research institutions such as RMCA, RBINS and the National Institute of Criminalistics and Criminology, but also universities, botanical and zoological gardens, and regional institutes dedicated to medical, agricultural, and conservation research. It aims at stimulating collaborative research by providing a discussion, training and exchange forum with respect to DNA barcoding. Therefore, BeBoL is dedicated to, amongst others, organizing meetings, workshops, symposia, and congresses.

So far, one of the most visible achievements of BeBoL was the organization of the “Third European Conference for the Barcode of Life, Brussels, 17–20 September 2012” (ECBOL3), under the thematic flag “Barcoding of organisms of policy concern”. This theme was chosen in view of the increasing interest of governments, decision makers, public authorities, law enforcement entities and private companies in DNA barcoding as a practical and reliable identification tool. As such the conference also provided an overview of DNA barcoding as an example of “applied taxonomy”. This formula appeared to be attractive since ECBOL3, which took place in the Royal Flemish Academy of Belgium for Science and the Arts (KVAB), was attended by about 120 researchers from Europe and beyond.

Although it was originally not planned to publish congress proceedings of ECBOL3, many participants felt that it nevertheless would be a great opportunity to produce a collection of DNA barcoding papers that emanated either from the congress or from BeBoL partners. Hence, it was decided to do so and to use this occasion to implement the unique possibilities offered by the open-access journal ZooKeys, a trend-setting taxonomic publication forum that extends papers with a whole series of extra features such as XML marking up and linking/transferring taxonomic data to ZooBank, GBIF, EOL, PLAZI and WikiSpecies. As such, ZooKeys illustrates the future of publishing freely accessible (big) biodiversity data in a global community, by what is often referred to as data hosting and the development of data publishing workflows. Therefore, ZooKeys is one of the core elements in the EU funded 7^th^ Framework Program ViBRANT (Virtual Biodiversity Research and Access Network for Taxonomy). This network has also been instrumental for JEMU, BeBoL and ECBOL3, since these initiatives have organized their communities by means of scratchpads, one of the core products of ViBRANT.

This special ZooKeys issue on DNA barcoding is hence the fruit of all the aforementioned efforts. It deals with a wide array of animal and plant taxa, and aims at demonstrating various aspects of DNA barcoding, including fundamental biodiversity research, applications, methodological issues, software, and limitations. Therefore, we hope that this issue may provide a modest, but lasting contribution to the already vast literature on DNA barcoding.

Brussels, December 11^th^, 2013

On behalf of BeBoL

This issue was realized with the support of:

**Figure:**